# RNA polymerase II subunit modulation during viral infection and cellular stress

**DOI:** 10.1016/j.coviro.2022.101259

**Published:** 2022-09-23

**Authors:** Leah Gulyas, Britt A Glaunsinger

**Affiliations:** 1 Department of Plant and Microbial Biology, University of California, Berkeley, CA 94720, USA; 2 Department of Molecular and Cell Biology, University of California, Berkeley, CA 94720, USA; 3 Howard Hughes Medical Institute, University of California, Berkeley, CA 94709, USA

## Abstract

Control of gene expression, including transcription, is central in dictating the outcome of viral infection. One of the profound alterations induced by viruses is modification to the integrity and function of eukaryotic RNA polymerase II (Pol II). Here, we discuss how infection perturbs the Pol II complex by altering subunit phosphorylation and turnover, as well as how cellular genotoxic stress (e.g. DNA damage) elicits similar outcomes. By highlighting emerging parallels and differences in Pol II control during viral infection and abiotic stress, we hope to bolster identification of pathways that target Pol II and regulate the transcriptome.

## Introduction

The battle between a virus and its infected cell is one that centers on the control of gene expression. On the cellular side, these are the responses that drive antiviral signaling, while the ability of a virus to block those signals and simultaneously redirect gene expression machinery toward viral genes can define whether an infection will amplify or be restricted. Viruses thus frequently manipulate transcriptional regulators such as activators and repressors, as well as chromatin structure [1]. However, for protein-coding genes, broad alterations in gene expression can also be achieved by targeting RNA polymerase II (Pol II) directly.

Pol II is a 12-subunit complex that is responsible for mRNA transcription in eukaryotic cells. Its activity relies on accessory proteins such as general transcription factors (GTFs), TATA-associated factors, and the mediator complex; the ensemble of this machinery is collectively referred to as the Pol II holoenzyme (for a thorough review, see Osman and Cramer 2020) [2]. Polymerase components and accessory factors are commonly restricted during infection to attenuate host gene expression, often of innate immune and antiviral genes [3–6]. Viral targeting of Pol II subunits is also a feature shared with cellular genotoxic stress such as UV damage, where it is thought to be critical for dampening the accumulation of transcripts encoding oncoproteins and transcription factors [7•].

Despite the prevalence of stress-linked Pol II abundance changes, much remains to be discovered about the mechanistic basis of these phenotypes, particularly in the case of infection. However, emerging evidence suggests that there is at least some conservation in the pathways that control Pol II during viral and nonviral stress, which we explore herein to create a framework to guide future studies on how stress modulates the cellular transcriptional landscape.

## Viruses alter RNA polymerase II phosphorylation and subunit abundance

### RNA Polymerase II phosphorylation

**Figure 1:**
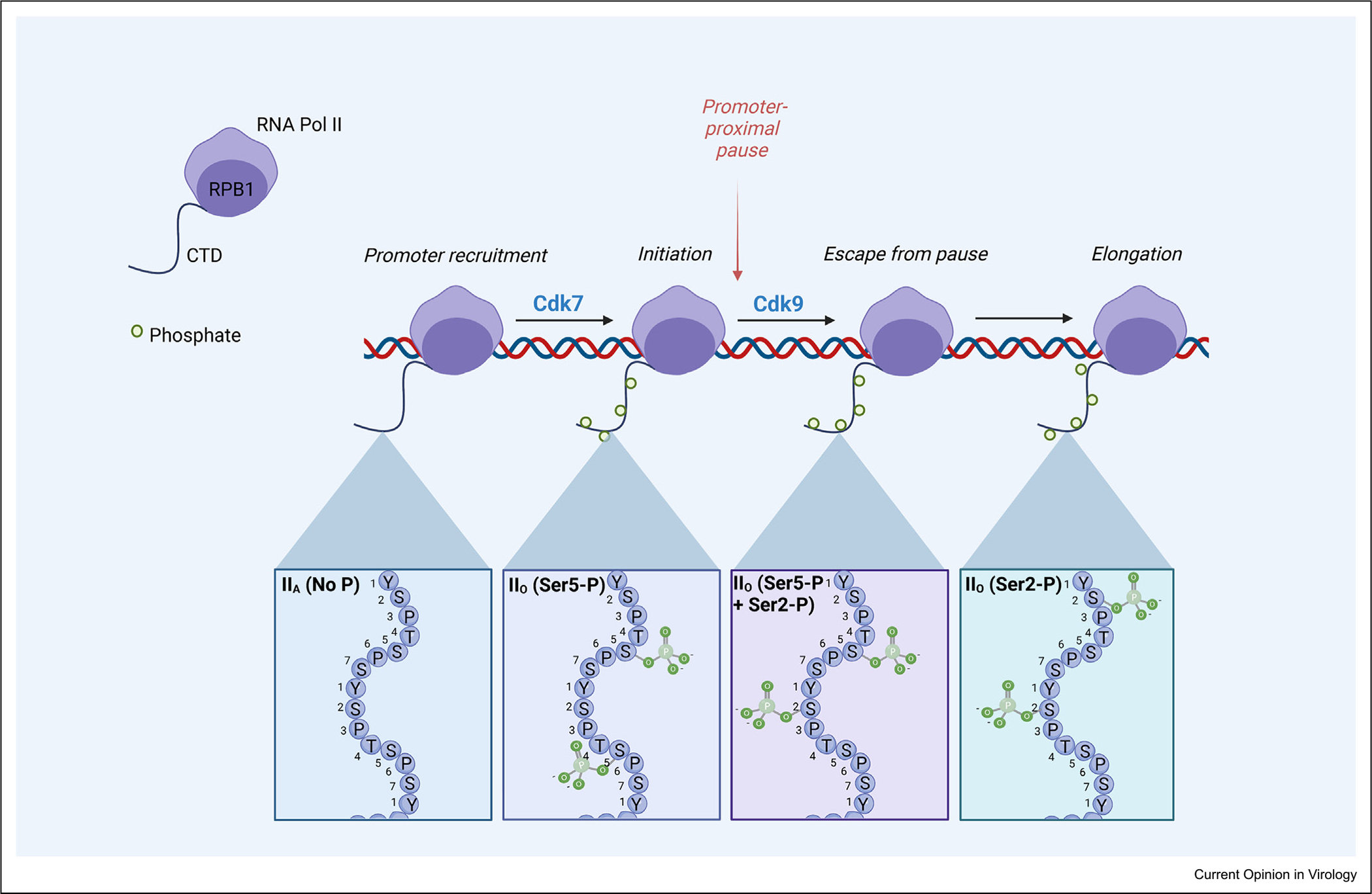
Pol II transcription is regulated by phosphorylation of the Rpb1 C-terminal domain. Hypophosphorylated Pol II (“II_A_”) is recruited to promoters and the heptad repeats of the CTD become phosphorylated (P) at Ser5 residues (primarily by kinase activity of the Cdk7 subunit of TFIIH) to generate hyperphosphorylated Pol II (II_O_). Following transcription initiation, Pol II temporarily stalls at a promoter-proximal pause site. Phosphorylation of Ser2 CTD residues by Cdk9 of the positive transcription elongation factor b permits Pol-II escape into the gene body, commencing transcriptional elongation. This is accompanied by a progressive dephosphorylation of Ser5 residues. Regulatory phosphorylation at Ser7 and modifications to other residues provide additional transcriptional control but are not discussed in this review. See Laybourn and Dahmus [51], Heidemann et al. [52], and Osman and Cramer [2] for more information.

Regulation of the Pol II holoenzyme occurs through post-translational modifications that influence the stages of transcription (Figure 1). Rpb1 (also known as POLR2A in mammals), the largest and primary catalytic subunit of Pol II, undergoes a well-defined phosphorylation cycle at different serine residues of its 52 heptad repeat-containing carboxyl-terminal domain (CTD) to control transcription initiation, pausing, and elongation [2]. Such regulatory phosphorylation is used during transcription of viral as well as host genes, although nuclear-replicating DNA viruses can induce noncanonical modifications to Rpb1. The best- characterized example of this occurs during herpes simplex virus (HSV-1) infection, in which expression of the immediate early protein ICP22 is sufficient to induce a general loss of serine-2-phosphorylated (Ser2-P) Pol II [8,9]. This is hypothesized to occur through ICP22 engaging in an inhibitory interaction with the CTD kinase Cdk9 and other elongation factors [9,10]. Ser2-P is associated with transcriptional elongation, and chromatin immunoprecipitation experiments confirmed that ICP22-mediated repression of Ser2-P reduces Pol II occupancy through the gene body [10]. HSV-1 ICP22 and UL13 expression also induce an intermediately phosphorylated species of Pol II (II_I_), composed mainly of serine 5-phosphorylated (Ser5-P), a modification that is usually associated with transcription initiation and splicing [11–13]. Pol II species-specific imaging experiments suggest that Pol II_I_ and hypophosphorylated Pol II are present in viral replication compartments following recruitment by the IE protein ICP27, while Ser2-P Pol II is specifically excluded or degraded [8,14]. That said, some minimal level of Ser2-P may be required for viral transcription since canonical Pol II regulatory stages still occur on the HSV-1 genome [15].

Other herpesviruses such as cytomegalovirus also alter CTD phosphorylation even in the absence of Pol II depletion [16]. While the broader implications of phosphorylation alterations have yet to be fully explored, they are likely to play an important role as HSV-1 infection has profound impacts on host transcription, ranging from decreased Pol II occupancy on host chromatin [17] to activation and repression of different host Pol II-dependent genes [18], as well as impairment of proper Pol II transcription termination [19]. Notably, Pol II phosphorylation changes are not limited to herpesviruses, as some cytoplasmic RNA viruses (e.g., Bunyamwera virus) similarly prevent host gene expression by blocking Ser2-P [20].

### Turnover of RNA polymerase II subunits

In addition to reshaping post-translational modifications of Pol II, diverse viruses impact Pol II activity by downregulating the protein abundance of Pol II subunits, reminiscent of several stress responses (discussed later). For RNA viruses that encode their own RNA-dependent RNA polymerase (RdRp), targeting Pol II enables selective dampening of host gene expression because viral genes are transcribed by the RdRp. Old World alphaviruses, including Sindbis, Chikungunya, and Semliki Forest virus, potentiate a rapid disappearance of vertebrate Rpb1 that involves proteasomal degradation. This is linked to viral nonstructural protein 2 (nsP2), as viruses unable to cleave and release nsP2 do not elicit Rpb1 degradation, and nsP2 expressed from a noncytopathic replication vector is sufficient to reduce Rpb1 levels [21]. *Bunyavirale* NS proteins also broadly impede host mRNA transcription to offset interferon induction [20,22••], and one member of this family, La Crosse virus (LACV), achieves this partly through NSs-directed Rpb1 degradation [22••]. Similarly, in addition to disrupting Pol II transcription by cap-snatching, the subunits of the Influenza A RdRp potentiate depletion of unphosphorylated Rpb1 through physical interactions of the two polymerases [23,24], while causing significant transcription-termination defects [25].

Some DNA viruses achieve similar outcomes, despite many of these viruses relying on host Pol II for viral transcription. This paradox is presumably resolved by recruitment of the remaining Pol II to viral lytic replication compartments by the open, comparatively nucleosome-depleted chromatin environment of the viral genome [26,27]. As mentioned above, HSV-1 infection prevents phosphorylation of Rpb1 Ser2 but also downregulates overall Rpb1 levels, suggesting Ser2-P-modified Rpb1 may additionally be degraded during infection [14]. While most studies have focused on Rpb1 abundance, whole cell proteomics-based measurements during murine gammaherpesvirus Murine herpesvirus 68 (MHV68) infection revealed depletion of six Pol II complex-specific subunits in addition to Rpb1 [28••]. Whether other viruses shown to reduce Rpb1 levels, including the gammaherpesvirus Kaposi’s sarcoma-associated herpesvirus [26], similarly deplete other Pol II subunits, remains an open question.

## Mechanisms of RNA polymerase II subunit depletion

**Figure 2:**
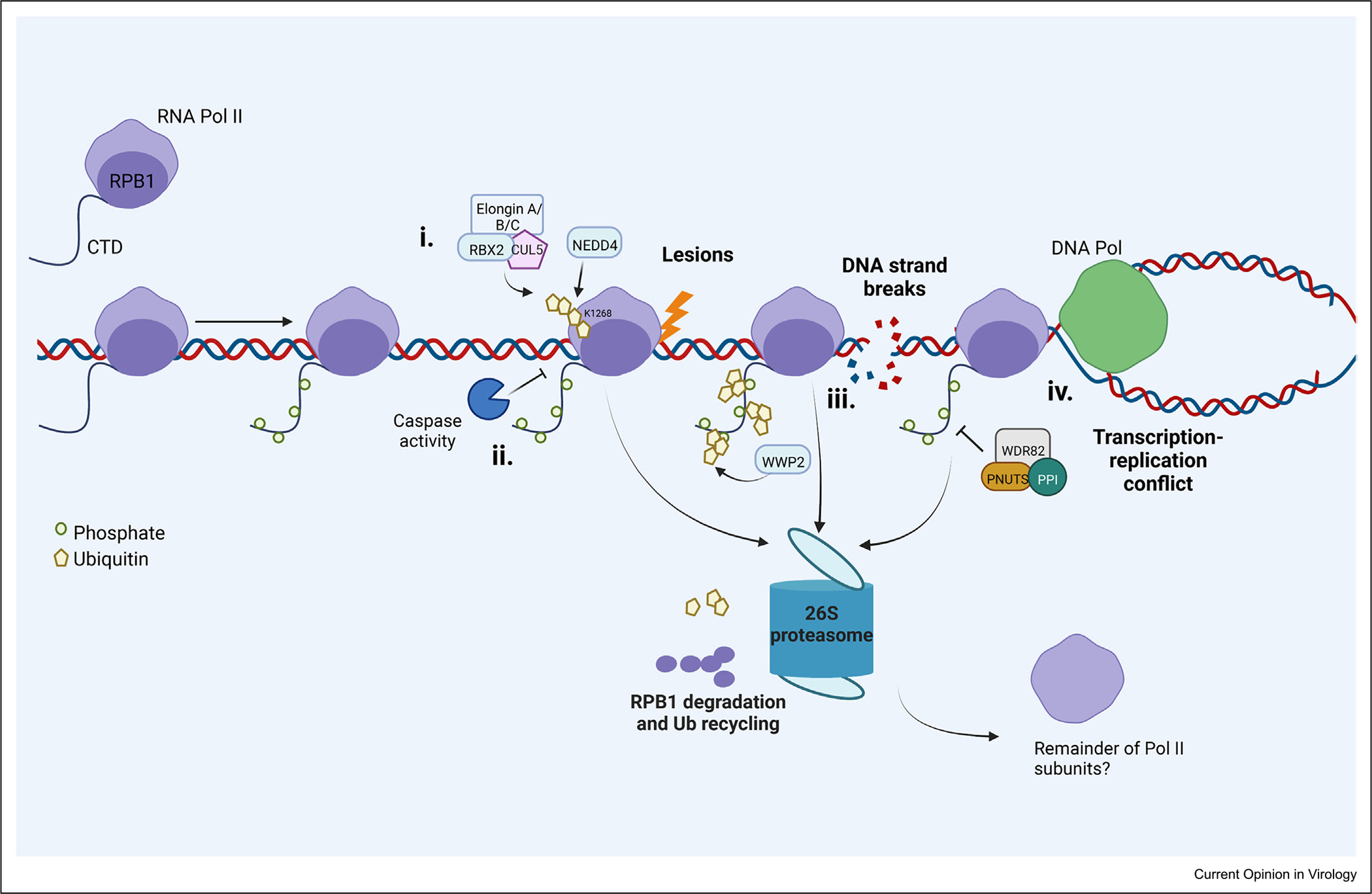
DNA damage response pathways target Rpb1 for degradation. **(i)** If UV lesions are sensed during transcriptional elongation, paused Pol II can become ubiquitylated on residue K1268 by Nedd4, and subsequent ubiquitylation by the cullin-RING E3 ligase complex Elongin A/B/C–RBX2–CUL5, leading to proteasomal degradation. **(ii)** Alternatively, caspase activity can proteolyze Pol II. **(iii)** dsDNA strand breaks cause WWP2-mediated Rpb1 CTD ubiquitylation and proteasomal degradation. **(iv)** Transcription–replication conflicts are resolved in part by Wdr82/PNUTS-PP1 CTD phosphatase activity followed by proteasomal degradation.

**Figure 3:**
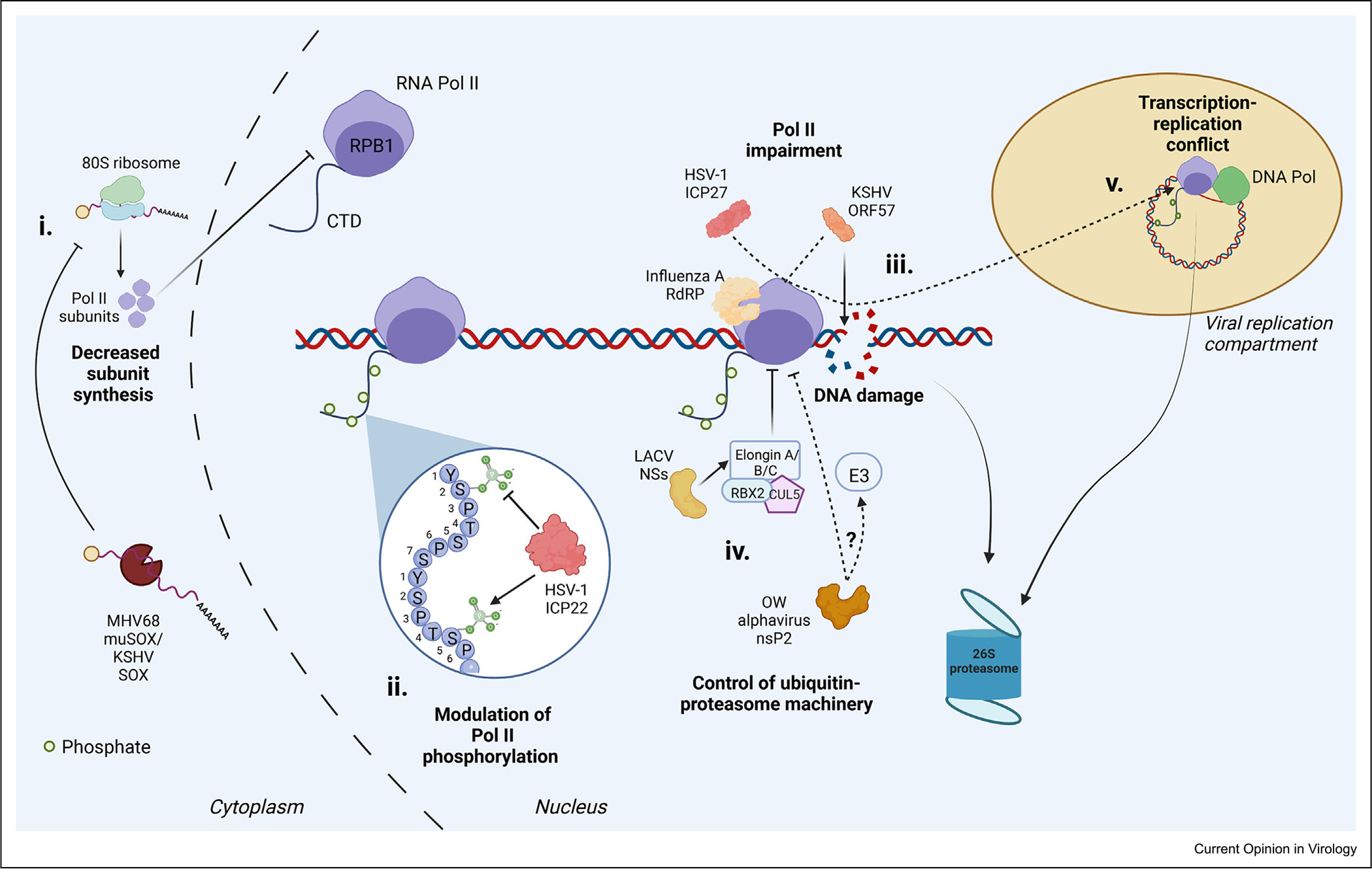
Model for potential viral interactions with and outcomes for Pol-II machinery. **(i)** Decreased mRNA abundance due to viral nuclease activity or decreased transcription results in decreased subunit synthesis for (and possibly destabilization of) Pol II complexes. **(ii)** Pol II CTD heptad phosphorylation is modulated by viral proteins to promote specific forms such as Ser5-P in HSV-1 and prevent Ser2-P needed for host transcript elongation. **(iii)** Pol II transcription can be impeded by interactions between viral proteins and Pol II machinery, some of which are expressed by dsDNA viruses to recruit machinery to vDNA in replication compartments. Stalling may license DDR machinery to interact with and promote Rpb1 ubiquitylation. **(iv)** Alternatively, viral-induced DNA damage may cause DDR-induced removal of Rpb1, or viral proteins could directly interface with ubiquitin–proteasome machinery to target Rpb1 for degradation. Unknown mechanisms may also be involved, such as induction of apoptosis and caspase-mediated subunit cleavage. **(v)** The polymerase collision model of transcription–replication conflicts on viral genomes could also promote proteasomal Rpb1 turnover.

Removal of Pol II occurs with many stressors, often resulting in proteasomal degradation of Rpb1 (Figure 2). Here, we overlay these strategies with those occurring during viral infection to highlight potential mechanistic conservation, as well as areas of divergence (Figure 3).

### The ubiquitin–proteasome system directs RNA polymerase II turnover in response to DNA damage

The best-defined mechanism of Pol II subunit turnover occurs in the context of the DNA damage response (DDR) in uninfected cells (summarized in Figure 2). UV light and other genotoxic agents induce base lesions or double-strand breaks that cause Pol II to stall upon encountering damage [29]. These bulky lesions initiate transcription-coupled nucleotide excision repair, in which Pol II accessory factors such as the XPB subunit of TFIIH permit Pol II backtracking for repair machinery to access the site [30]. However, if the repair is insufficient for transcription to restart, Pol II is instead removed via a proteasome-mediated pathway, whereby mammalian E3 ligases Nedd4 and the Elongin A/B/C–Cul5–Rbx2 complex sequentially ubiquitylate Rpb1 to target it for extraction from the holoenzyme and degradation [31,32]. In yeast, another Pol II subunit, Rpb9, is reported to promote the ubiquitylation process [33], as well as the CCR4–Not complex, which regulates Pol II elongation and mRNA decay [34,35], suggesting multiple points of control.

Double-strand breaks also prompt Rpb1 proteasomal degradation, though in a mechanistically distinct pathway involving WWP2-mediated ubiquitylation of lysine residues within the Rpb1 CTD [36]. Threats to genome integrity incurred during transcription–replication conflicts are similarly dealt with by Pol II proteasomal degradation, first requiring Wdr82/PNUTS–PP1 dephosphorylation of Ser5-P on the CTD [37•]. In summary, while the intermediary factors are distinct, each of these pathways converges on ubiquitin-mediated proteasomal degradation of Rpb1.

### Viral parallels with the DNA damage-activated pathways of RNA polymerase II turnover

Like the DNA damage response pathways, Pol II depletion during RNA virus infection involves the ubiquitin–proteasome pathway [21,22••,^24^], as proteasome inhibitors at least partially rescue Rpb1 protein levels [21,22••]. The clearest link to the DDR machinery comes from LACV infection, where siRNA-mediated depletion of the Elongin A/B/C complex partially recovers Rpb1 levels [22••]. La Crosse virus also upregulates DDR markers, mirroring UV damage-induced Rpb1 clearance [6,22••]. However, the observation that proteasomal inhibition rescues Rpb1 levels more robustly than Elongin knockdown suggests that additional unidentified E3 ligases participate in La Crosse NSs-induced Rpb1 degradation [22••]. Regardless, one notable feature is the extremely rapid Rpb1 depletion, which occurs as early as 1h post La Crosse virus infection [22••].

Pol II depletion by the nsP2 protease from Old World alphaviruses also occurs via the proteasome rather than by direct cleavage, as proteasomal inhibition rescues Rpb1 levels substantially, whereas mutation of the nsP2 protease domain does not [21]. Similar to a DDR-like pathway, transcriptional inhibition with the DNA intercalator actinomycin D also rescues Rpb1 levels. Unlike the DDR pathway however, preventing transcriptional elongation by inhibiting Ser2-P does not restore Rpb1 abundance [21]. It is possible that nsP2 recruits DDR or other proteasomal machinery directly to Pol II, or that its DNA binding activity might promote Pol II turnover prior to Pol II elongation.

Many viruses interface with DDR machinery in the host cell by promoting host DNA damage, preventing viral DNA damage or recognition, and facilitating viral DNA replication and repair [38–40] — each of which could initiate DDR pathways that remove Pol II. As noted previously, the proteasome-dependent reduction of Pol II in HSV-1 infection appears most pronounced for the elongating Ser2-P form, which would align with DDR pathway activation [14]. Furthermore, transcription–replication conflicts and the resulting DNA breaks may create an environment conducive to prominent Pol II turnover during DNA virus genome replication [14].

A major limitation to our understanding of the specific turnover pathways involved in infection-induced Pol II depletion is the lack of evidence implicating specific E3 ligases (even Elongin C knockdown with LACV infection contributes only a subtle rescue). More unbiased screening approaches to determine the contributions of different E3s or ubiquitylation residues are needed to reveal links to DDR pathways as well as identify alternate or new pathways. If these mechanisms were inhibited in the presence of virus, this could also permit assessment of the functional implications of Pol II destruction for both virus and host. Furthermore, given that proteasome inhibition yields incomplete rescue of Pol II abundance during viral infection, regulation of Pol II abundance likely relies on the convergence of multiple processes.

### Complex interactions and mRNA decay in RNA polymerase II turnover

Rpb1 turnover during UV DNA damage does not result in co-depletion of other complex subunits in yeast, suggesting that individual subunits may be independently rather than interdependently regulated [29,33]. That said, Rpb1 degradation appears to potentiate nuclear depletion of at least Rpb2 and Rpb3 in mammalian cells, as was demonstrated in the context of Rpb1 depletion by fusion to an auxin-inducible degron (AID) tag [41]. It is therefore notable that lytic MHV68 infection causes depletion of 7 of the 12 Pol II subunits, and that all of the depleted subunits are Pol II specific, whereas the spared subunits are shared with Pol I and Pol III [28••]. One possibility is that MHV68 primarily targets one subunit, and the others co-deplete, for example if their stability is dependent on complex formation. This could explain why Pol II-specific subunits are degraded; if lack of a limiting Pol II subunit decreases Pol II construction, shared subunits may instead bind Pol I and III complexes. Interestingly, MHV68 infection upregulates transcriptional activity from some Pol III promoters [42–44]. It is tempting to speculate that a loss of Pol II-specific subunits contributes to the increased Pol III transcription during infection. This would be consistent with a recent study from Gerber et al. [41], in which Pol II depletion by AID-tagging or preventing Pol II transcription initiation upregulated Pol III-dependent tRNA synthesis.

Other possible mechanisms are that MHV68 targets multiple subunits independently for protein turnover, or that select protein subunit(s) are targeted directly while others deplete due to a failure to replenish the protein pool upon degradation of their mRNA. In this regard, widespread mRNA degradation is a well-established feature of lytic gammaherpesvirus infection and Pol II subunit levels are not reduced upon infection with an MHV68 mutant that is deficient for mRNA decay [28••,^45^,^46^]. Virus-induced mRNA decay could also play a role if one subunit of the complex is stoichiometrically limiting for Pol II formation; in this case, the levels of a short-lived protein would be reliant on its mRNA abundance.

MHV68-induced mRNA decay also prompts the redistribution of RNA binding proteins to the nucleus, which is both necessary and sufficient to induce host transcriptional repression [47]. While the mechanistic role of these nuclear-translocated RNA binding proteins in dampening transcription has not been identified, one hypothesis is that they compete with resident nuclear proteins for nascent RNA binding in a manner that phenocopies Pol II stalling and potentiates its turnover. Interestingly, shuttling of cytoplasmic poly(A) binding protein to the nucleus and transcriptional repression are phenotypes associated both with apoptosis [48] and UV damage [49]. Though the kinetics differ among these examples, the underlying biological similarities may speak to deeper and more complex links between a variety of stressors.

## Perspectives and conclusions

The Pol II holoenzyme is critical for maintaining and regulating the cellular transcriptome. The consequences of shifting Pol II activity and abundance are particularly important for stress responses; thus, understanding how this occurs in the context of viral infection is crucial for predicting host–virus interactions. Data suggest that some protein-level Pol II adjustments occur via conserved pathways during infection and genotoxic conditions, but there are major gaps in our knowledge of how these are activated, as well as the functional consequences.

While many viral Pol II depletion strategies seem to be at least partially dependent on proteasomal degradation, in general, the E3-mediated pathways leading to this outcome have not been identified. Regulation of Pol II abundance likely results from the combined effects of converging pathways — individually testing the few E3s implicated in other stress responses probably cannot provide a complete picture. Moreover, not all Pol II turnover occurs via the proteasome. UV radiation is reported to induce a less prominent, caspase-mediated cleavage event near the Rpb1 CTD [50], and another subunit, Rpb2, also shows a modest protein reduction during apoptosis [48]. This multi-subunit regulation highlights the importance of more comprehensively examining the Pol II complex during cellular stress; Rpb1 is often the only subunit that is rigorously inspected, though other Pol II subunits and holoenzyme components such as GTFs play pivotal roles in complex activity.

Screening approaches are needed to flesh out the pathways involved in infection-induced changes to Pol II. Identification of these pathway components could additionally provide more targeted tools beyond inhibitors of the ubiquitin–proteasome system or transcription (which are often toxic and have pleotropic effects) to interrogate the impact of Pol II depletion or modification on the viral lifecycle. In this way, we could identify new players similarly involved in Pol II regulation during abiotic stress. While much remains to be discovered about the dynamics of Pol II regulation, viral research has and will continue to provide critical insight into this fundamental cellular process.
